# Different Influences of Negative and Neutral Emotional Interference on Working Memory in Trait Anxiety

**DOI:** 10.3389/fpsyg.2021.570552

**Published:** 2021-03-31

**Authors:** Huifang Yang, Junqing Li, Xifu Zheng

**Affiliations:** ^1^Department of Psychology, School of Education Sciences, Lingnan Normal University, Zhanjiang, China; ^2^Guangdong Provincial Key Laboratory of Development and Education for Special Needs Children, Lingnan Normal University, Zhanjiang, China; ^3^Key Laboratory of Psychological Assessment and Rehabilitation for Exceptional Children, Lingnan Normal University, Zhanjiang, China; ^4^Department of Physical Education Sciences, Lingnan Normal University, Zhanjiang, China; ^5^School of Psychology, South China Normal University, Guangzhou, China

**Keywords:** emotion, working memory, ERP, LPP, trait anxiety

## Abstract

To examine the interaction of working memory (WM) type with emotional interference in trait anxiety, event-related potentials were measured in a combined WM and emotional task. Participants completed a delayed matching-to-sample task of WM, and emotional pictures were presented during the maintenance interval. The results indicated that negative affect interfered with spatial WM; task-related changes in amplitude were observed in the late positive potential (LPP) and slow waves in both the high and low anxiety groups. We also found an interaction among WM type, emotion, and trait anxiety such that participants with high levels of trait anxiety showed an opposite neural response to verbal and spatial WM tasks compared with individuals with low trait anxiety during the sustained brain activity involved in processing negative or neutral pictures in the delay phase. Our results increase our understanding of the influence of emotions on recognition and the vulnerability of those with trait anxiety to emotional stimuli.

## Highlights

► This study examined the effects of emotional distraction on working memory in individuals with trait anxiety.
► Participants with high trait anxiety showed an opposite neural response to verbal and spatial working memory tasks compared with participants with low trait anxiety.
► Negative distraction interfered with spatial working memory.

## Introduction

### Cognitive Control in Anxiety

Cognitive models of anxiety suggest that impaired cognitive control plays a critical role in the development and maintenance of anxiety (Song et al., 2017). Cognitive control is the ability to arrange thought and action in accordance with task-related goals and consists of a variety of distinct executive processes that include attention control, maintenance of working memory (WM), and inhibition control (Braver, 2012). In the process of cognition, the inhibition is defined as the mechanism of preventing the irrelevant information from entering the WM or eliminating the irrelevant information from the WM. A variety of studies demonstrated that individuals with trait anxiety tend to show attentional bias toward threatening or negative information (MacLeod et al., 2019), potentially leading to increased threat detection or anxious experiences. This vulnerability increases the degree of interference from threatening or negative information, which is unrelated to ongoing task, and decreases the ability of inhibitory control (Zhang et al., 2019). Such impaired cognitive control in trait-anxious individuals may lead to the entering of threat-related information into their WM, increasing their worry and other anxiety-related cognitions that interfere with ongoing task.

### Emotional Interference and WM in Anxiety

Within the context of cognitive control, emotional interference is the emotionally salient stimuli that may potentially impair cognitive task (e.g., perception and WM) and can compromise the ability to complete tasks requiring cognitive control (Song et al., 2017). Emotional interference impairs WM as the emotional information tends to capture and reallocate cognitive resources (Shafer and Dolcos, 2012). Empirical research has provided strong evidence to support the existence that negative stimuli are more difficult to ignore than non-affective interference. Cognitive control mechanisms may be recruited to mitigate the interfering effect of such distractors and improve WM performance (Pacios et al., 2020). Interference from distracting stimuli occurs when inhibition of it fails. To be specific, in the trait-anxious group, this interference may reduce the attentional resources used for the WM task, impairing the WM task performance and maintaining the level of anxiety (Zhang et al., 2019). While an emotional impairment effect on WM performance has been shown in several studies at the behavioral and the neural levels (Kennedy et al., 2018; Okruszek et al., 2018; Zhang et al., 2019; Pacios et al., 2020), less is known about how emotional interference influences WM in trait-anxious individuals so far.

Anxiety is always accompanied by changes in cognitive processing, and the effects of anxiety on cognitive performance may be mediated by their effects on WM (Eysenck and Calvo, 1992; Owens et al., 2014). Increasing evidence indicates that the performance of anxious individuals is more easily impaired by threat-related interference than is that of individuals without anxiety (Grosdemange et al., 2015). Anxiety is also associated with inactivity of the neural circuit involved in cognitive control (Sari et al., 2016), which has been described as a defect in cognitive control. As attentional control is the key function of the central executive (Eysenck et al., 2007; Berggren and Derakshan, 2013), recognition of the impact of anxiety on attention processing is crucial for understanding how anxiety influences cognitive performance. According the attentional control theory of anxiety, trait anxiety impairs the ability of attentional control (Eysenck and Derakshan, 2011) and the inhibition process of WM. Previous evidence has shown that an inhibitory control deficit in individuals with anxiety interferes with the inhibition process of WM (Zhang et al., 2019), causing attention resources that are captured by the stimulus of emotional significance, and the inhibition of irrelevant information fails.

WM system is composed of three advanced cognitive operations: the phonological loop dealing with verbal information, the visuospatial sketchpad for processing non-verbal visual and spatial information, and the central executive for control. The central executive acts as more of an attention system—particularly in maintaining task goals and reducing interference from distraction (Moran, 2016). From the perspective of evolutionary psychology, emotional information is important for survival. Generally, in the context of emotional interference and dual-task paradigms, task-independent emotional disturbance injures task-relevant performance (Shafer and Dolcos, 2012). It is not clear, however, how this initial processing of task-irrelevant distracting emotional information influences WM task in trait anxiety. The present study addressed these questions.

## The Prior Study

Previous studies have examined the influence of emotional interference on WM. Imaging studies with a delayed matching-to-sample task demonstrated that negative emotional disturbance presented during the delay period hindered performance (Dolcos and McCarthy, 2006; Dolcos et al., 2006). They found evidence that negative interference decreased the active maintenance of goal-relevant information-related brain activation in the dorsolateral prefrontal cortex (dlPFC), whereas there was an increased emotional processing–related brain activation in the ventrolateral PFC and amygdala (Dolcos and McCarthy, 2006; Iordan and Dolcos, 2015). As one important executive process, maintenance of WM task is the function of cognitive control. Emotional interference signaling potential danger can lead to cognitive conflict and impair the ability to maintain WM task requiring cognitive control (Banich et al., 2009). Recently, lots of studies have suggested a common neural circuitry underlying cognitive–emotional conflict resolution (Pessoa, 2008; Song et al., 2017). Some brain regions such as dlPFC are related to cognitive control, as well as emotion processes (Okon-Singer et al., 2015).

Prior research has demonstrated that distracting tasks may reduce emotion-related physiological responses, such as the event-related (ERP) components of late positive potential (LPP) and slow waves, which are sensitive to affective stimuli. Early components of LPP have been interpreted as responses to attentional capture, recognition, and stimulus evaluation (Donchin and Coles, 1988). With a dual task, Schupp and colleagues (1997) found that the perceptual processes demanding attentional resources can reduce the attentional resources usable for subsequent processing, resulting in an attenuated magnitude of the LPP to the latter probes (Schupp et al., 1997). However, a dissociation between the affective modulation of the LPP and attention (emotional interference) has been recently described in previous studies on repeated exposure (Codispoti et al., 2016; Micucci et al., 2020). It has been demonstrated that attentional capture by emotion waned after only a few presentations of the same distractor, whereas the LPP amplitude was still enhanced for emotional, compared with neutral, distractors despite stimulus repetition. Similarly, distractor frequency reduced attentional capture by emotional distractors, whereas the frequency effect on the affective modulation of the LPP was not reduced in either study, suggesting that emotional stimuli continued to engage the motivational system even when the emotional interference on the primary task was suppressed. Positive slow potentials reflect increased sustained attention (Cuthbert et al., 2000) and may have a role in memory storage (Donchin and Coles, 1988). Ruchkin and colleagues (1988) have proposed that positive slow waves vary with the amount of information maintained in memory (Ruchkin et al., 1988). Thus, given the functional sensitivity of LPPs and slow waves, as well as the excellent temporal resolution of ERPs, LPPs and slow waves may be ideal for studying the time course of the impact of emotional interference on WM in anxious individuals during the maintenance phase.

## The Present Study

The aim of the present study was to examine the interaction of WM type with emotional interference in trait anxiety subjects with the ERP method. Based on existing research reviewed above, we hypothesized that the effects of anxiety on the neural correlates of WM are attributable to a specific component function of WM. Second, the influence of anxiety on the neural correlation of WM attributes to the different valences of the emotional distractors.

## Methods

### Ethics

Participants gave written informed consent to participate in the study. And the written informed consent was approved by the local ethics committee.

### Participants

Three hundred nineteen undergraduate students completed the Chinese version of the Trait Anxiety Inventory (TAI) (Spielberger et al., 1983) approximately 6 weeks prior to the study. The 319 participants never participated in any research run by our laboratory before. Following previous studies (Ansari and Derakshan, 2011), we selected participants with high scores (ranged from 44 to 62) and low scores (ranged from 28 to 34) on trait anxiety to further consideration. We used the method of random sampling, that is, from the high-score and low-score subjects, randomly selected eligible subjects, and called them to ask if they would like to participate in the experiment. From these groups, we randomly invited 43 volunteers (aged 19–23 years, all right-handed, with normal or corrected-to-normal vision) to participate in the experiment. In order to match for sex, we try to keep gender balance in each group. Two participants were excluded from the experiment because they did not finish the task seriously. There were 41 effective participants in the two groups. Among them, 21 participants (11 females, mean TAI score = 46.38, SD = 6.26) were in the high trait anxiety group (HA group), and 20 participants (13 females, mean TAI score = 31.35, SD = 6.27) were in the low trait anxiety group (LA group). Although we want to control the sex ratio, most of the students in our university are female students, not enough male participants in mass screening to achieve gender balance in the LA group. All participants were proficient in reading the Latin alphabet. All participants were paid for their participation in the study.

### Stimulus Materials

The emotional stimuli were 120 pictures consisting of 60 aversive pictures and 60 neutral pictures, which were selected from the International Affective Picture System (Lang et al., 2005). According to the independent affective ratings given by 20 undergraduate students using a nine-point scale, the aversive pictures were significantly more negative in valence [1.7 ± 1.12 vs. 5.04 ± 1.26; *t*(58) = −68.43, *P* < 0.001] and more highly arousing [7.25 ± 1.72 vs. 3.93 ± 1.81; *t*(58) = 46.12, *P* < 0.001] than were the neutral pictures.

The WM materials were 24 letters from the Latin alphabet. To ensure that the physical characteristics of the two types of stimuli are completely identical, the stimulus sets for the verbal and spatial task were the same (Li et al., 2010). The instructions for them were different. Instruction for verbal task was to remember the name of letter and then judge the consistency between the preceding letter and the later letter, ignoring the letter's location. However, the instruction of spatial task required participants to remember and judge only the location of the letter.

### Procedure

The participants were seated in an electronically isolated, sound- and light-attenuated room and viewed a computer monitor from a distance of 75 cm.

**Figure 1:**
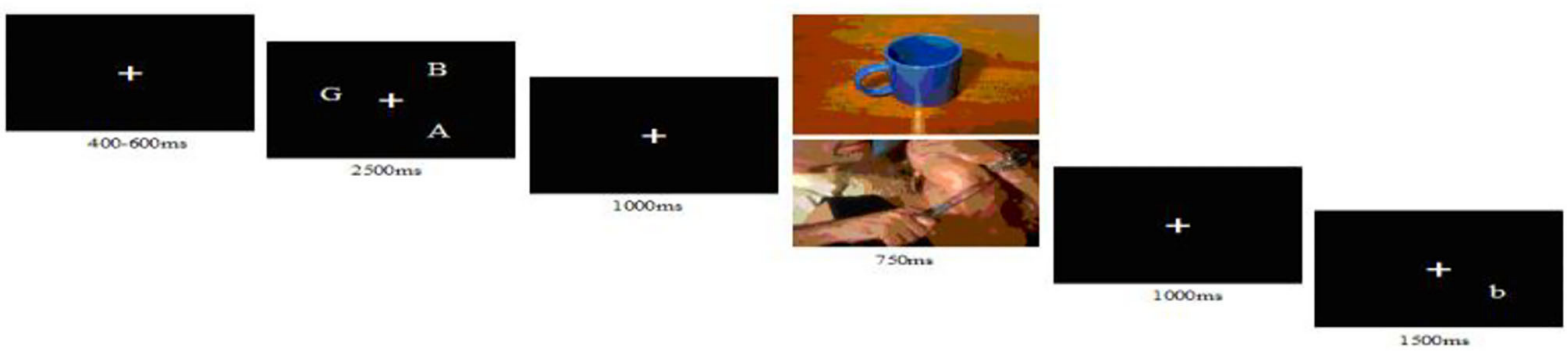
The sequence of events in a trial. The picture was neutral or negative.

First, a white fixation point “+” appeared in the center of the black background for 400–600 ms. Three uppercase letters then appeared at anywhere around the “+” for 2,500 ms. The letter stimuli occupied 3–5° of visual angle on the visual midline. The participants were instructed to remember the three letters, followed by a white “+” for 1,000 ms. After the presentation of a picture (neutral or negative) for 750 ms, a white “+” appeared for 1,000 ms. Finally, a small letter appeared at 1 of the 12 points corresponding to the clock (Figure 1). Participants were instructed to judge whether the small letter was the same as the one of the preceding uppercase letters. To ensure that the physical characteristics of the two types of stimuli were completely identical, the stimulus sets for verbal and spatial tasks are the same, but the difference lies in the guidance. In the verbal task, participants were instructed to judge whether the name of the letter in the probe phase was the same as or different from that of the letter in the target phase and to ignore the letter's location. The spatial task required participants to remember and judge only the location of the letter.

Participants completed a short training session consisted of 12 stimuli, followed by two formal sessions. Only one kind of emotional picture appeared in one session. Sessions were separated by a 5-min interval. In order to avoid emotional disturbance, the neutral session appeared before the aversive session. Each session was composed of two blocks, one verbal and one spatial, appearing in random order. Each block included 60 trials, resulting in a total of 240 trials, which appeared completely randomized. There was a brief rest when participants finished 30 trials.

### Electroencephalogram Recording and Analysis

Electroencephalograms (EEGs) were recorded from 64 scalp electrodes located in standard 10/20 electrode positions embedded in an elastic cap recording device (NeuroScan version 4.3 system). All electrodes were referenced to the M2 (right mastoid) and then re-referenced offline to the average of M1 (left mastoid) and M2. EEGs were recorded with a 0.01–100-Hz bandpass filter and 1,000-Hz sampling rate. Electrode impedances were always kept below 5 kΩ. Vertical electro-oculogram (EOG) recording electrodes were positioned above and below the left eye, and horizontal EOG recording electrodes were positioned at the outer canthi of both eyes. Each epoch was filtered with a 24-Hz low-pass filter. Trials with various artifacts were rejected using a criterion of ±100 μV. The ERPs were averaged for trials with correct responses.

The EEG was segmented for each trial beginning 100 ms before to 1,000 ms after the picture onset. Based on previous studies (Hajcak et al., 2009; MacNamara et al., 2011), the ERP components were scored by averaging the amplitudes of the three time windows following picture onset, including the early LPP (296–356 ms), late LPP (452–512 ms), and slow waves (600–760 ms).

Mean error rates and reaction times (RTs) were entered into a 2 × 2 × 2 mixed analysis of variance (ANOVA), with task type (spatial/verbal) and valence (negative/neutral) as within-subject factors and group (LA/HA) as between-subject factor. Besides, all electrophysiological data were analyzed by repeated-measures ANOVA also including electrode (FC/CP/P/PO) and laterality (left/midline/right) as within-subject factors. Greenhouse–Geisser adjustments to the degrees of freedom were performed.

## Results

### Behavioral Results

#### Accuracy

ANOVA results revealed a main effect of emotion [*F*(1, 39) = 17.3, *p* < 0.001, η^2^ = 0.31]; the accuracy (ACC) under the negative-emotion condition (91.5 ± 0.6%) was higher than that under the neutral-emotion condition (86 ± 1.5%). The task type × emotion interaction was significant, *F*(1, 39) = 4.74, *p* < 0.05, η^2^ = 0.11. A further simple-effect test reflected that verbal WM (85.58 ± 0.7%) was significantly more accurate than was spatial WM (82.34 ± 0.9%) under the negative-emotion condition [*F*(1, 39) = 23.47, *p* < 0.001, η^2^ = 0.38]. Additionally, the accuracy of verbal WM was higher under the neutral-emotion condition (94 ± 0.7%) than it was under the negative-emotion condition (85.6 ± 2.3%). ACC values for each condition in each group are shown in Table 1.

**Table 1:** Basic descriptive statistics of ACC and reaction time (RT) in both groups.

**Task type**	**Interference type**	**ACC**		**RT**	
		**LA**	**HA**	**LA**	**HA**
Verbal	Neutral	86.35 ± 13.39%	84.81 ± 15.91%	803.98 ± 125	821.34 ± 114.95
	Negative	94.2 ± 4.4%	93.76 ± 4.36%	759.54 ± 98.37	759.67 ± 74.06
Spatial	Neutral	86.8 ± 9.62%	86.1 ± 6.2%	717.56 ± 101.85	756.33 ± 112.77
	Negative	89.85 ± 6.27%	88.24 ± 5.16%	679.01 ± 83.6	703.51 ± 100.03

#### Reaction Times

ANOVA results revealed a main effect of task type [*F*(1, 39) = 47.48, *p* < 0.001, η^2^ = 0.55]; the RTs for spatial WM tasks (786.13 ± 15 ms) were longer than those for verbal WM tasks (714.11 ± 14.83 ms). The main effect of emotion was significant [*F*(1, 39) = 26.2, *p* < 0.001, η^2^ = 0.4], with the RTs under the negative-emotion condition (774.81 ± 16.25 ms) being longer than those under the neutral-emotion condition (725.43 ± 13.15 ms). RT values for each condition in each group are shown in Table 1.

### ERP Results

#### Early LPP

**Figure 2:**
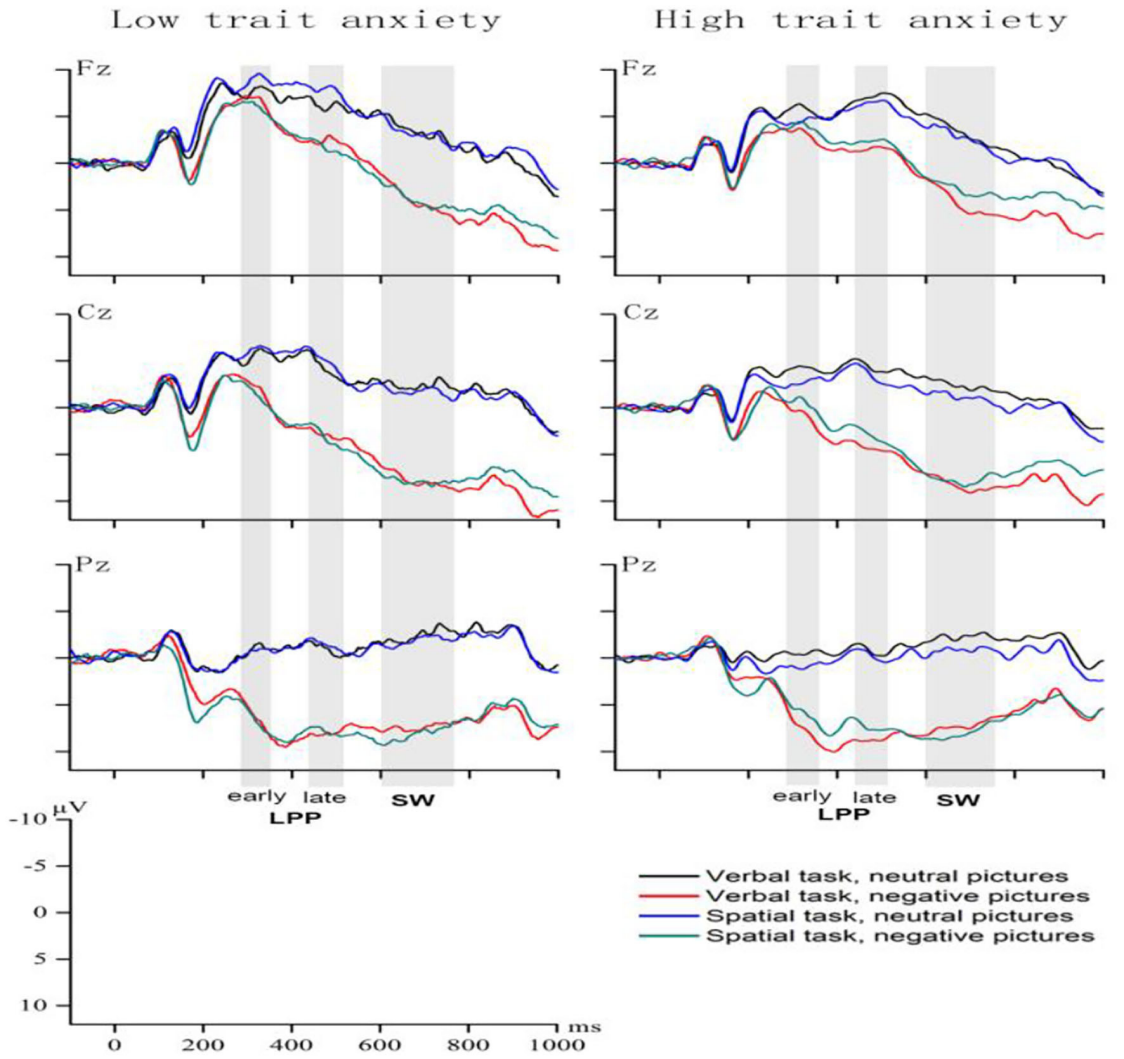
Grand-average waveforms in the LA and HA groups under the four conditions.

The ANOVA conducted on early LPP (Figure 2) showed a main effect of emotion [*F*(1, 39) = 104.95, *p* < 0.001, η^2^ = 0.73] and electrode [*F*(3, 117) = 119.3, *p* < 0.001, η^2^ = 0.75], with the amplitudes of early LPPs higher under the negative-emotion (5.17 ± 0.64 μV) than the neutral-emotion (0.5 ± 0.41 μV), and the amplitude of the parietal–occipital electrodes highest (6.43 ± 0.72 μV) and that of the frontal–central electrodes lowest (−3.74 ± 0.53 μV). The main effect of laterality was significant, *F*(2, 78) = 66.85, *p* < 0.001, η^2^ = 0.63. According to the amplitude from large to small: left side (3.93 ± 0.52 μV) > right side (3.72 ± 0.47 μV) > middle line position (0.86 ± 0.55 μV).

The task type × emotion interaction, *F*(1, 39) = 6.23, *p* < 0.05, η^2^ = 0.14, was followed by simple effect analysis for verbal WM and spatial WM, and then neutral-emotion and negative-emotion, respectively. The negative pictures elicited larger early LPPs compared to the neutral pictures in both the verbal [(5.41 ± 0.69) vs. (0.26 ± 0.46) μV] and the spatial [(4.93 ± 0.64) vs. (0.74 ± 0.42) μV] tasks.

The task type × emotion × group interaction was significant, *F*(1, 39) = 14.03, *p* < 0.001, η^2^ = 0.27. The simple effect of group for neutral emotion × spatial WM interaction was significant, and the HA group showed larger early LPP (1.65 ± 0.59 μV) amplitude than did the LA group (−0.17 ± 0.61 μV). The simple effect of task type was significant for the HA group under the neutral [*F*(1, 39) = 8.23, *p* < 0.01, η^2^ = 0.17] and negative-emotion [*F* (1, 39) = 5.96, *p* < 0.05, η^2^ = 0.13]. When the HA group viewed neutral pictures during the maintenance phase, the pictures elicited higher early LPP amplitude during spatial WM task (1.65 ± 0.59 μV) than the verbal WM task (0.45 ± 0.64 μV). In contrast, after the HA group viewed negative pictures, the pictures elicited higher early LPP amplitude during the verbal WM task (6.01 ± 0.95 μV) than the spatial WM task (4.8 ± 0.89 μV). No significant differences were found for these other group comparisons.

#### Late LPP

Regarding late LPP amplitude (Figure 2), analysis showed a main effect of emotion, *F*(1, 39) = 149.6, *p* < 0.001, η^2^ = 0.8, the negative emotion elicited a higher amplitude of the late LPP (4.34 ± 0.64 μV) compared to the neutral emotion (−1.69 ± 0.38 μV).

The task type × emotion × group interaction was significant, *F*(1, 39) = 6.45, *p* < 0.05, η^2^ = 0.14. The interaction effect was examined by comparing the effect of group for each combination of task type and emotional condition. Under both of the two WM task conditions, the late LPP elicited under the negative-emotion was larger than the neutral-emotion in the HA group in comparison to the LA group. The simple main effect of emotion was significant for the HA group under the verbal [*F*(1, 39) = 82.27, *p* < 0.001, η2 = 0.68] and spatial WM tasks [*F* (1, 39) = 44.31, *p* < 0.001, η2 = 0.53]. During verbal WM task, the negative pictures elicited higher late LPP amplitude (4.99 ± 0.95 μV) than the neutral pictures (−1.87 ± 0.64 μV). Similarly, during spatial WM task, the negative pictures elicited higher late LPP amplitude (3.84 ± 0.92 μV) than the neutral pictures (−1.37 ± 0.53 μV). No significant differences were found for these other group comparisons.

The main effect of laterality was significant, *F*(2, 38) = 37.53, *p* < 0.001, η^2^ = 0.66. According to the amplitude from large to small: left side (1.88 ± 0.47 μV) > right side (1.71 ± 0.44 μV) > middle line position (0.38 ± 0.52 μV).

The interaction effect of task type × laterality × group was significant, *F*(2, 38) = 4.51, *p* < 0.05, η^2^ = 0.19. Further simple effect test showed that the simple main effect of laterality was significant in verbal [*F*(2, 38) = 18.71, *p* < 0.001, η^2^ = 0. 5] and spatial [*F*(2, 38) = 20.48, *p* < 0.001, η^2^ = 0.52] in the LA group. In the low anxiety group, the amplitude of LPP in the left side of the brain was the largest both in verbal (2.03 ± 0.76 μV) and spatial (1.61 ± 0.44 μV) tasks. In the HA group, The simple main effect of laterality was significant both in the verbal [*F*(2, 38) = 17.36, *p* < 0.001, η^2^ = 0.48] and spatial[*F*(2, 38) = 12.99, *p* < 0.001, η^2^ = 0.41, respectively] WM tasks in the HA group. In the HA group, the LPP amplitude in the left side of the brain (2.31 ± 0.74 μV) was the largest in verbal tasks, similar to the low anxiety group. In contrast to the LA group, the LPP amplitude in the right side of the brain was the largest (1.71 ± 0.62 μV) in spatial tasks in the HA group.

#### Slow Wave

The ANOVA conducted on slow wave showed a main effect of emotional state [*F*(1, 39) = 178.23, *p* < 0.001, η^2^ = 0.82], with slow-wave amplitude under the negative emotion (4.67 ± 0.31 μV) higher than the neutral emotion (1.89 ± 0.32 μV). The main effect of laterality was also significant [*F*(2, 78) = 13.66, *p* < 0.001, η^2^ = 0.26], and the amplitude of the left lateral electrodes was the highest (2.56 ± 0.42 μV).

We found a marginally significant task type × electrode × group interaction effect, *F*(3, 117) = 2.62, *p* = 0.055, η^2^ = 0.06. We observed a significant difference [*F*(1, 39) = 8. 08, *p* < 0.01, η^2^ = 0.17] between the amplitudes elicited by the interference pictures during the verbal and spatial tasks in the HA group over only the parietal–occipital electrodes in comparison to the LA group, and the interference pictures elicited higher slow-wave amplitudes in the spatial (1.57 ± 0.59 μV) than in the verbal tasks (0.45 ± 0.66 μV).

The task type × emotion interaction was marginally significant, *F*(1, 39) = 3.55, *p* < 0.05, η^2^ = 0.08. The comparisons of the effects of emotional state for each task type revealed that negative emotion elicited higher amplitudes than neutral emotion in both the verbal and spatial tasks [*F*(1, 39) = 144.43, *p* < 0.001, η^2^ = 0.79; *F*(1, 39) = 142.02, *p* < 0.001, η^2^ =0.79] (Figure 2). During verbal WM task, the negative pictures elicited higher late LPP amplitude (5.77 ± 0.66 μV) than the neutral pictures (−1.7 ± 0.4 μV). Similarly, during spatial WM task, the negative pictures elicited higher late LPP amplitude (5.46 ± 0.68 μV) than the neutral pictures (−1.03 ± 0.35 μV).

#### Topographies of Difference Waves

**Figure 3:**
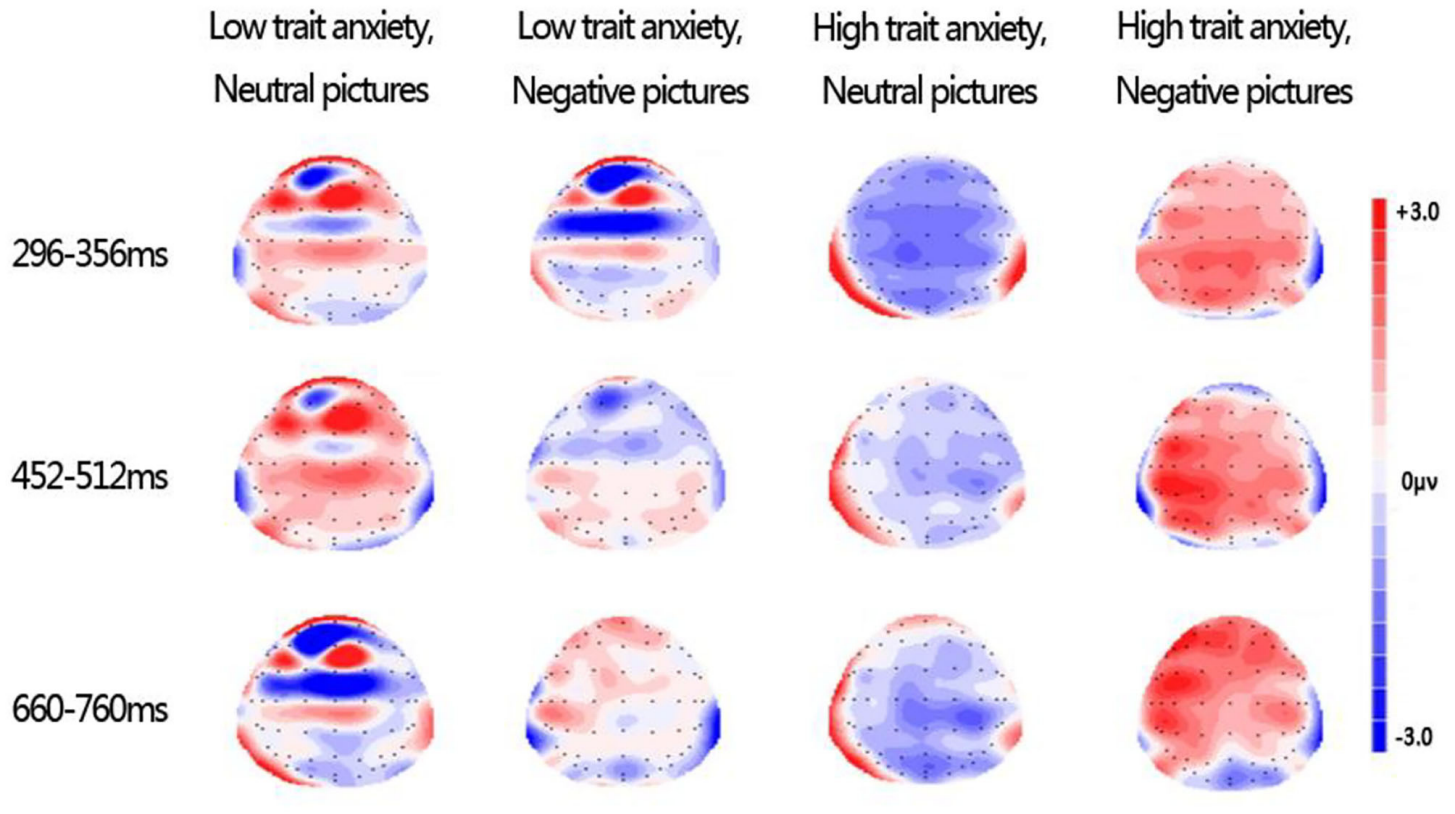
Topographies of difference waves formed by subtracting spatial trial ERPs from verbal trial ERPs during 296–356, 452–512, and 660–760 ms after picture onset.

Figure 3 illustrates the topography of difference waves (subtracting spatial trial ERPs from verbal trials) during 296–356, 452–512, and 660–760 ms following picture onset. Under negative conditions in the HA group, the topographies of difference waves were almost all red, indicating that the difference wave was almost positive, but under neutral conditions in the HA group, the topographies of difference waves were almost all blue, indicating that the difference wave was almost all negative. Combined with the calculation method of differential wave and Figure 3, we can know that, under the negative emotional interference, verbal WM induced a greater amplitude than spatial WM in the HA group; on the contrary, under the neutral emotional interference, the spatial WM induced a greater amplitude than the verbal WM amplitude. Compared with the LA group, the opposite neural pattern appeared only in the HA group. These results are consistent with the previous ERP results on early LPP. Thus, topography of difference waves reflected opposite neural response patterns in the HA group under the neutral and negative emotion conditions.

## Discussion

This study examined the effect of emotional interference on visual WM. The main aim was the interaction of WM type with emotional interference in trait anxiety.

Our findings demonstrate differential modulation of emotional interference in WM in trait anxiety. We report that participants with high trait anxiety showed a different neural response to verbal and spatial WM tasks. Compared with the low anxiety group, this reaction pattern appeared only in the high anxiety group. This provides direct electrophysiological measures of the disruptive effects of negative emotions and anxiety on WM. In other words, anxiety combined with emotion has an effect on WM. It is not only emotions that affect WM. This modulation depends not only on WM type but also on the value of emotional disturbance. Interestingly, the interference effect of emotional pictures on spatial WM in high trait anxiety shows opposite pattern.

We initially tested for the interference effect and found greater susceptibility to distraction due to interference by negative stimuli in spatial than in verbal WM. All participants made more errors and had longer RTs to the probe for spatial compared with verbal material during the maintenance delay under the negative-emotion conditions. The behavioral data showed that interference by negative emotions impaired spatial WM. A number of studies have found poorer spatial WM performance during interference by negative affect (Dolcos and McCarthy, 2006; Okruszek et al., 2018). The authors suggested that this effect is due to competition for limited visuospatial attention resources (Lavric et al., 2003; Shackman et al., 2006).

Also, task performance was no different between the LA and HA groups. Previous similar study also observed no impaired performance in the HA group (Zhang et al., 2019). We suggest that this lack of impairment in the group may be due to the following reasons: first, university students are likely high achieving and less representative of general population; second, HA participants may take strategies to complete the task (Espuny et al., 2018; Zhang et al., 2019) and avoid loss when the task performance may be affected by the interference.

The findings suggest that the neural responses involved in the processes underpinning sustained brain activation during the delay phase of verbal and spatial WM tasks may be opposite in individuals with high trait anxiety, depending on whether they are responding to negative or to neutral pictures. In contrast, individuals with LA may not have opposite neural responses. Specifically, participants with high trait anxiety had higher early LPP amplitudes in response to neutral pictures during the maintenance phase of the spatial WM task relative to the verbal WM task. In contrast, participants with high trait anxiety had higher earlier LPP amplitudes in response to negative pictures during the maintenance phase of the verbal WM task relative to the spatial WM task.

Why was HA associated with opposite neural responses during sustained brain activation during the delay phase of verbal and spatial WM tasks involving negative vs. neutral affective pictures? This question can be answered in terms of LPP and the processing mechanisms underpinning spatial WM. The early components of LPPs represented attentional capture, recognition, and stimulus evaluation (Donchin and Coles, 1988). From the perspective of survival, threat-related negative pictures elicited larger deflections in early LPPs (Böcker et al., 2001). The competition for limited visuospatial attention resources between spatial WM and negative pictures (Erk et al., 2007) leads to the preceding negative pictures during the maintenance phase capture more attention resources, results in less attention resources left for the process of spatial WM. As mentioned previously, several studies support a two-process account of the LPP modulation during affective picture viewing, in which the resistant core reflects a mandatory process implied in the detection of stimulus motivational significance, whereas attentional allocation to emotional stimuli wanes with stimulus repetition (Ferrari et al., 2013, 2016, 2020; Codispoti et al., 2016; Micucci et al., 2020).

Our ERP results indicated that the amplitudes of the early LPPs elicited by the spatial WM task were larger than those elicited by the verbal WM task in the HA group during the maintenance phase following the neutral pictures. This supports the assumption that attentional resources are required for spatial WM but not necessarily for verbal WM (Li et al., 2010). Additionally, according to attentional control theory (Eysenck et al., 2007), maintenance of a low level of anxiety requires HA individuals to invest more attentional resources in spatial WM. Thus, the amplitude of LPP during the maintaining phase of the spatial WM task was much higher than it was during the verbal WM task under the neutral-emotion condition.

In contrast, after seeing negative pictures, the amplitude of the early LPP of the HA group was higher for the verbal compared with the spatial WM task. Donchin and Coles (1988) interpreted the early components of LPP as attentional capture, recognition, and stimulus evaluation. This supports the assumption that the spatial WM task consumes such a large portion of the visuospatial attentional resources that less neural activity remains for attentional capture by negative pictures in the context of negative interference. Thus, because of the limited attentional resources available for processing negative interference pictures, the amplitudes of the LPP evoked by negative pictures were lower during the spatial WM compared with the verbal WM task during the maintenance phase.

The results revealed such an effect in that the late LPP and slow-wave components also showed task-related amplitude changes in the HA and LA groups. The LPP extends throughout the entire duration of picture presentation, indexing increased sustained attention to emotional stimuli (Foti et al., 2009; Hajcak et al., 2009). We also found a prominent interaction effect of group and unilateral brain region during WM-related brain activation. During the verbal WM task, both groups showed the highest LPP amplitudes in the left brain region. However, the HA group showed opposite patterns of brain activation during different WM tasks. In the HA group, the LPP amplitude in the left brain region was highest during the verbal WM task, whereas the LPP amplitude in the right brain region was highest during the spatial WM task. As LPP is modulated by the dynamic allocation of attention, and spatial WM involving perceptual processing needs more attention in the right hemisphere (Simon-Thomas et al., 2005), this pattern suggests enhanced attention to interference pictures during the maintenance phase (Hajcak et al., 2010) of spatial WM task in the HA group in comparison to the LA group. Furthermore, the higher amplitude of a slow-wave pattern reflects the allocation of a particular type of additional resources as well as additional effort to a task (Rosler et al., 1997). Because some of the resources of HA participants are already allocated to anxiety person, these participants must exert additional effort in the presence of an interference stimulus (i.e., the amplitude of their slow waves is higher). Overall, because the HA group devoted excessive attentional resources to emotional pictures, the resources available in the right hemisphere were limited, detracting from the attentional resources available for spatial WM (Schupp et al., 1997). As a result, when interference stimuli appear, the WM of individuals with HA is impaired because such stimuli cannot be effectively inhibited.

Individuals with high trait anxiety demonstrate affective bias toward aversive or threat-related stimuli (Wilson and MacLeod, 2003), which typically reduces attention to a current task. Thus, the probability that processing resources shifts from task-related WM to task-unrelated emotional stimuli increases. Additionally, anxiety is associated with reduced influence of the goal-directed attentional system (Corbetta and Shulman, 2002) involved in the top-down control of attention as well as increased influence of the stimulus-driven attentional system (Corbetta and Shulman, 2002) involved in the bottom-up control of attention. This causes a reduction in attentional control and dysfunction related to inhibition and shifting. As a result, when interference stimuli appear, the WM of individuals with HA is impaired because such stimuli cannot be effectively inhibited.

Our behavioral and ERP results show HA individuals demonstrating disparate neural processing yet equivalent performance to LA individuals, indicating that impaired task performance is not a characteristic of HA individuals. Instead, HA individuals tend to be “strategic” in that they avoid mistakes in completing the task. The real-world implications of our results are that impaired task performance is not the only evidence to judge whether an individual is highly anxious, and EEG data should also be combined to judge.

Our findings may contribute to the comprehension of WM in trait anxiety. On the one hand, our findings demonstrate the generality of impaired cognitive control in HA individuals, not only in attentional control, but also in the inhibition of negative interference from WM. Our results extend prior observations of inhibition control failures at the stage of maintenance (Qi et al., 2014; Zhang et al., 2019) and add to previous indications that deficits in inhibitory control of task-irrelevant interference are present in HA individuals. On the other hand, our results provide electrophysiological evidence of the interaction between WM and emotional interference, and the interaction is modulated by anxiety level. The previous study by Zhang et al. (2019) showed that HA individuals is linked to deficits of inhibitory control that can consume attentional resources, and emotional interference additionally affects the late processing stage and increases the required cognitive control. Our findings, together with previous investigation (Song et al., 2017; Okruszek et al., 2018; Pacios et al., 2020), point to the failure of cognitive control in trait anxiety.

The present study has some limitations. A limitation of the WM task was the use of letters for both the spatial and verbal WM conditions. This is because during the spatial condition it would have been more difficult to inhibit the automatic human nature of reading the visual stimuli than it would be to inhibit spatial location (and this extra cognitive load/attentional resources may partially explain the difference in RTs between the two conditions) and the higher early LPP amplitude during spatial WM task compared to the verbal WM task in the HA group.

In conclusion, our findings demonstrate that negative emotional distractors have differential effects on neural attentional processing for individuals with high vs. low trait anxiety, suggestive of possible compensatory/resilience mechanisms at work to allow comparative task scores in individuals with high trait anxiety. Our results suggest that such mechanisms have important effects on the performance of cognitive tasks and that the kind of effect depends on the type of the task (verbal or spatial), on the level of trait anxiety (higher levels of trait anxiety are associated with more interference), and on the type of emotional interference (neutral or negative). Our results contribute to our understanding of the influence of emotions on recognition performance and trait anxiety, which are associated with increased susceptibility to emotional disruptions.

## Data Availability Statement

The datasets presented in this article are not readily available because the data also forms part of the ongoing study. Requests to access the datasets should be directed to Huifang yang, 954147041@qq.com.

## Ethics Statement

The studies involving human participants were reviewed and approved by the Ethics Committee of Lingnan Normal University. The patients/participants provided their written informed consent to participate in this study. Written informed consent was obtained from all participants for the publication of any potentially identifiable data included in this article.

## Author Contributions

HY: experiment design. JL: data collection. XZ: major revision of the manuscript. All authors contributed to the article and approved the submitted version.

## Conflict of Interest

The authors declare that the research was conducted in the absence of any commercial or financial relationships that could be construed as a potential conflict of interest.
