# Supplementary material for: Different Influences of Negative and Neutral Emotional Interference on Working Memory in Trait Anxiety
Source: Front Psychol. 2021 Mar 31;12:570552. doi: 10.3389/fpsyg.2021.570552 (PMC8044409; doi:10.3389/fpsyg.2021.570552)
Supplement: Supplementary file 1 [file Data_Sheet_1.ZIP › Summarized data/ERP data/Grand-average waveforms data.doc/Grand-average waveforms in the LA groups under the four conditions.doc]

**Grand-average waveforms in the LA groups under the four conditions**

11:verbal tasks, neutral pictures

12:verbal tasks, negative pictures

21: spatial tasks, neutral pictures

22：spatial tasks, negative pictures
