# Supplementary material for: Different Influences of Negative and Neutral Emotional Interference on Working Memory in Trait Anxiety
Source: Front Psychol. 2021 Mar 31;12:570552. doi: 10.3389/fpsyg.2021.570552 (PMC8044409; doi:10.3389/fpsyg.2021.570552)
Supplement: Supplementary file 1 [file Data_Sheet_1.ZIP › Summarized data/ERP data/Grand-average waveforms data.doc/Illustration.doc]

What does the data account for

G11_1(20).avg represent grand-average waveforms of verbal tasks and neutral pictures in the LA groups(20 participants)

G11_2(21).avg represent grand-average waveforms of verbal tasks and neutral pictures in the HA groups(21 participants)

G12_1(20).avg represent grand-average waveforms of verbal tasks and negative pictures in the LA groups(20 participants)

G12_2(21).avg represent grand-average waveforms of verbal tasks and negative pictures in the HA groups(21 participants)

G21_1(20).avg represent grand-average waveforms of spatial tasks and neutral pictures in the LA groups(20 participants)

G21_2(21).avg represent grand-average waveforms of spatial tasks and neutral pictures in the HA groups(21 participants)

G22_1(20).avg represent grand-average waveforms of spatial tasks and negative pictures in the LA groups(20 participants)

G22_2(21).avg represent grand-average waveforms of spatial tasks and negative pictures in the HA groups(21 participants)
