# Supplementary material for: Different Influences of Negative and Neutral Emotional Interference on Working Memory in Trait Anxiety
Source: Front Psychol. 2021 Mar 31;12:570552. doi: 10.3389/fpsyg.2021.570552 (PMC8044409; doi:10.3389/fpsyg.2021.570552)
Supplement: Supplementary file 1 [file Data_Sheet_1.ZIP › Summarized data/ERP data/grand-average difference waves/Illustration.doc.doc]

What does the data account for

G11-21_1(20).avg represent grand-average difference waves of verbal tasks and neutral pictures

formed by subtracting spatial and neutral trial ERPs from verbal tasks and neutral trial ERPs in the LA groups(20 participants).

G11-21_2(21).avg represent grand-average difference waves of verbal tasks and neutral pictures

formed by subtracting spatial and neutral trial ERPs from verbal tasks and neutral trial ERPs in the HA groups(21 participants).

G12-22_1(20).avg represent grand-average difference waves of verbal tasks and negative pictures

formed by subtracting spatial and negative trial ERPs from verbal tasks and negative trial ERPs in the LA groups(20 participants).

G12-22_2(21).avg represent grand-average difference waves of verbal tasks and negative pictures formed by subtracting spatial and negative trial ERPs from verbal tasks and negative trial ERPs in the HA groups(21 participants).
